# Quantum-enhanced interferometer using Kerr squeezing

**DOI:** 10.1515/nanoph-2023-0032

**Published:** 2023-04-10

**Authors:** Nikolay Kalinin, Thomas Dirmeier, Arseny A. Sorokin, Elena A. Anashkina, Luis L. Sánchez-Soto, Joel F. Corney, Gerd Leuchs, Alexey V. Andrianov

**Affiliations:** Max Planck Institute for the Science of Light, 91058 Erlangen, Germany; Nonlinear Dynamics and Optics Division, Institute of Applied Physics of the Russian Academy of Sciences, Nizhny Novgorod 603950, Russia; Physik Department, Friedrich-Alexander-Universität Erlangen-Nürnberg, 91058 Erlangen, Germany; Advanced School of General and Applied Physics, Lobachevsky State University of Nizhny Novgorod, Nizhny Novgorod 603022, Russia; Departamento de Óptica, Facultad de Física, Universidad Complutense, Madrid 28040, Spain; School of Mathematics and Physics, University of Queensland, Brisbane, QLD 4072, Australia

**Keywords:** fiber squeezing, interferometric sensitivity, optical Kerr effect, squeezed light

## Abstract

One of the prime applications of squeezed light is enhancing the sensitivity of an interferometer below the quantum shot-noise limit, but so far, no such experimental demonstration was reported when using the optical Kerr effect. In prior setups involving Kerr-squeezed light, the role of the interferometer was merely to characterize the noise pattern. The lack of such a demonstration was largely due to the cumbersome tilting of the squeezed ellipse in phase space. Here, we present the first experimental observation of phase-sensitivity enhancement in an interferometer using Kerr squeezing.

## 1 Introduction

When pushing the sensitivity of sensors to the limits, one will ultimately have to deal with quantum uncertainty or measurement projection noise. Braginsky [1] was the first to study this limitation in connection with early attempts to develop detectors for gravitational waves. This limitation is a general challenge in physics because some of the most sensitive measurements involve interference. Caves [2] and Loudon [3] understood the interplay of photon-counting noise or shot-noise limit (SNL) and light-pressure fluctuations in an interferometer giving rise to the standard quantum limit (SQL) [4], which led Caves to propose using squeezed light entering the usually dark input port of the interferometer for reducing the photon-counting error at lower powers of the driving laser [5]. Soon after, Yuen [6] and Unruh [7] established that one could even hope for beating the SQL, by correlating the two noise sources limiting the sensitivity. This was then confirmed by more detailed calculations [8], [9], [10].

Ultimately, the sensitivity of any interferometric measurement, not only in optics, is limited by the smallest structure in the corresponding phase space [11]. Squeezing the distribution in phase space by some nonlinear interaction is thus one way to increase the fundamental sensitivity. However, to achieve enhanced sensitivity, several conditions must be met. First, the arrangement of the interferometer and its detection scheme should maximize the sensitivity in a classical sense; i.e., the response of the measured quantities to changes in the phase difference between arms should be maximized. Simultaneously, the detection scheme should be arranged in such a way that the measured quantum noise is minimized; i.e., the detector is sensitive to the squeezed light quadrature only. In other words, the squeezed quadrature of the uncertainty distribution in phase space must be oriented parallel to the local trajectory along which the mean value moves in response to the phase change.

When the squeezed state is not centered at the origin in phase space, the situation is complicated because there are then three angles: one describing the mean value, another one describing the direction in which the structure is narrowest, and the third one corresponding to the orientation of the trajectory in phase space along which the average moves in response to the phase changes. In general, the squeezing angle is skewed with respect to the mean excitation, such as in the optical Kerr interaction. The Gross–Pitaevski equation [12, 13], describing cold atoms, contains a related nonlinearity, and elegant ways were found to deal with the skewed squeezing ellipse [14], [15], [16].

In the optical domain, due to the lack of a simple and obvious way to fulfill the above requirement, there has been no experimental demonstration so far for improving the sensitivity of an optical interferometer beyond the SNL using Kerr squeezing. We report the first such demonstration.

For these reasons, the reduction of photon-counting noise was first demonstrated using squeezed vacuum—with the mean value at the origin in phase space and an appropriate orientation of the squeezing ellipse—generated by parametric down-conversion (PDC) [17, 18]. The sensitivity enhancement is achieved by sending a laser beam into one input port (bright port) and squeezed vacuum into the other input port (dark port) with a proper adjustment of the relative phase. For a summary of squeezed light generation see Ref. [19]. In the meantime some experiments went way beyond the SNL and reached the ultimate Heisenberg limit [20], but only in the very low-power regime using entangled photons from PDC. For an application such as in gravitational-wave detection, one combines higher laser power and squeezed light to maximize the sensitivity [21]. The field has witnessed enormous progress and squeezed light is now applied to large-scale detectors [22, 23]. One of these large-scale interferometers even reached the SQL and observed the effect of radiation pressure [24] and further improvements in sensitivity can be expected using frequency-dependent squeezed light [25], [26], [27]. The squeezed vacuum used there is generated using degenerate PDC requiring phase matching, typically inside an optical resonator, and stabilization loops [19, 28].

A potentially more robust type of squeezing uses the optical Kerr effect, which occurs almost for free when light propagates through a fiber [29]. However, no interferometer has so far been made more sensitive beyond the SNL using this effect. A major reason is that the Kerr effect squeezes the quantum uncertainty of a coherent state so as to create amplitude-phase correlations resulting in the squeezed ellipse oriented under a skewed angle in phase space. As a result, neither the amplitude nor the phase quadrature becomes squeezed. Then it is challenging to arrange an interferometer such that at the same time (i) the detection scheme is sensitive to the tilted squeezed quadrature and (ii) it is maximally sensitive to the phase changes in the arm lengths (see Section 2 for more detail). The first theoretical proposals addressing challenge (i) suggested different ways to use an interferometer or a cavity to characterize Kerr squeezing [30], [31], [32], [33]. Several experimental groups implemented these and related interferometric schemes for measuring the Kerr-squeezed ellipse (for a review see e.g., [19]). But none of these interferometric setups were able to also address challenge (ii) and thus to demonstrate an improved sensitivity, as discussed in more detail below.

A further problem is thermal phase noise by forward Brillouin scattering acquired during light propagation through a room-temperature fiber. Recently, there was progress in generating sizeable, robust squeezing through the optical Kerr effect in a fiber minimizing room temperature phase noise [34].

## 2 Kerr-squeezing-improved interferometric sensitivity

Loudon [3] was the first to note the similarity between radiation pressure and the Kerr effect: both effects cause intensity-dependent phase shifts. Bondurant [35] and Pace et al. [10] studied theoretically the cancellation of radiation pressure by the Kerr effect. Consequently, radiation pressure can also lead to squeezing called ponderomotive squeezing. Further work included ponderomotive squeezing in design studies for improved interferometers [25, 36]. For references to experimental results on ponderomotive squeezing see Refs. [37, 38]. Still, there have been no experimental implementations using Kerr or ponderomotive squeezing to improve the phase sensitivity of an interferometer.

There was, however, one proposal by Shirasaki [39] to get around the challenge imposed by the skewed orientation of the Kerr squeezing ellipse and improve the sensitivity of an interferometer in the low-power regime, but it was not noticed much thus far. The results shown below were obtained along the lines of this proposal, which could have been straightforwardly implemented earlier, but its potential had not been recognized by experimental groups including ours until now.

**Figure 1:**
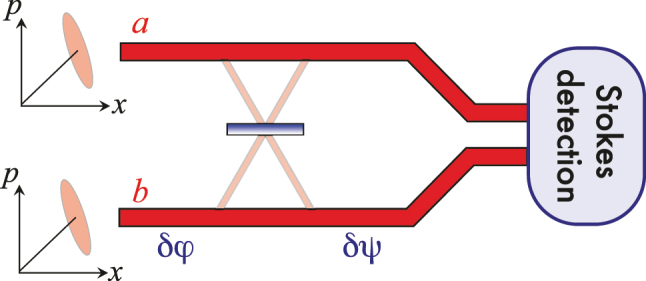
Two-rail sketch. Kerr-squeezed phase-coherent light beams of equal power are launched into rails *a* and *b*. The squeezing ellipses are tilted with respect to the amplitude quadrature and, without the central beam splitter, when measuring the Stokes parameter do not improve the interference sensitivity. On the contrary, it is much worse than it would be for coherent input states of similar power because the interferometer is sensitive to the relative phase and the Kerr squeezing increases the phase noise. Without the beam splitter, the phase differences before (*δφ*) and after (*δψ*) it are equivalent. With the beam splitter, a basis transformation is introduced that, if appropriately chosen, leads to a sensitivity beyond the SNL for *δψ*, but not for *δφ*.

So, we report the first experimental demonstration of interferometric sensitivity enhancement using the Kerr effect. Here, we concentrate on the low-power regime in which the photon-counting noise dominates, but the scheme can be extended (see the last paragraph in Section 3). The sensitivity enhancement using the Kerr effect is made possible by a modified setup that incorporates a basis change between the sources of nonlinearity and the measurement part of the interferometer. To emphasize the issue that this modification addresses, consider an interferometer with Kerr squeezed light in the two arms, as sketched in Figure 1. The initial beam splitter and the nonlinear Kerr interaction are not shown. If the two rails interfere in the Stokes parameter measuring detector without the central beam splitter in place, then the sensitivity to arm-length differences *δφ* is still much worse than shot noise because of the tilt of the squeezed ellipse and the large anti-squeezing. This reflects the situation discussed at the end of the previous Section.

The Stokes parameters are defined from the amplitude operators for the upper and the lower rails $\hat{a}$ and $\hat{b}$ , respectively, as

$$\begin{array}{cc} {\hat{S}}_{0} & ={\hat{a}}^{†}\hat{a}+{\hat{b}}^{†}\hat{b},\;{\hat{S}}_{1}={\hat{a}}^{†}\hat{a}-{\hat{b}}^{†}\hat{b},\\ {\hat{S}}_{2} & ={\hat{a}}^{†}\hat{b}+{\hat{b}}^{†}\hat{a},\;{\hat{S}}_{3}=i\left({\hat{b}}^{†}\hat{a}-{\hat{a}}^{†}\hat{b}\right). \end{array}$$

**Figure 2:**
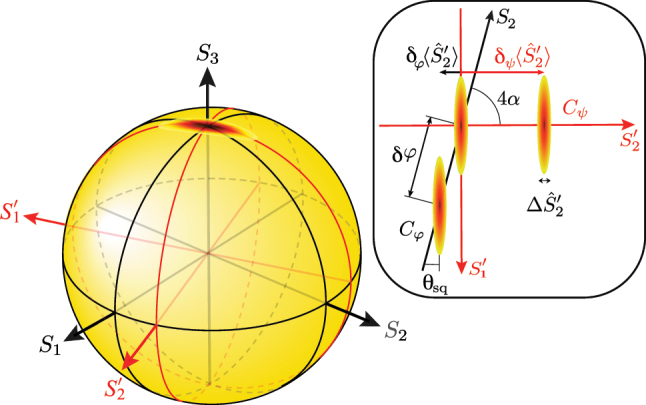
Poincaré-sphere representation of the state in the two-rail system just before interference in the Stokes parameter detector. Without the central beam splitter in Figure 1, the state just before the interference in the detector rotates along the black geodesic (great circle) in the *S*_2_–*S*_3_ plane as a function of optical arm length difference *δφ*. The joint effect of the squeezing in the two arms is a squeezed ellipsoid which is tilted with respect to the geodesic. The sensitivity is worse than the SNL because of the anti-squeezing. With the central beam splitter, the Stokes basis is changed such that the new $S_{1}^{\prime }$ axis has the same orientation as the major axis of the ellipsoid. Then further ‘downstream’, an arm length difference *δψ* lets the state rotate along the tilted red geodesic leading to a sensitivity improvement beyond the SNL. In the inset, we have a top view of the motion of the ellipsoid due to phase difference before (*δφ*) and after (*δψ*) the beam splitter.

To appreciate the full potential of this two-mode system, it is best described as an SU(2) interferometer using a variant of the Poincaré sphere [40], as shown in Figure 2. The two-rail system in Figure 1 evolves from left to right. Not shown is the first part of the interferometer, in which a coherent beam is split by a beam splitter and each of the split beams then undergoes a self-Kerr interaction. Suppose that, on the left side of Figure 1 the relative phase between the *a* and the *b* rails is such that the state is located on the *S*_3_ axis. In the Poincaré space, the squeezed quantum noise causes an ellipsoidal distribution of the state. Any arm length difference between rails *a* and *b* leads to a phase difference *δφ* corresponding to a rotation around the *S*_1_ axis that describes the photon-number difference between the two rails. The state trajectory is the black geodesic (great circle) and the Kerr squeezed ellipsoid is tilted with respect to this geodesic, as indicated in Figure 2. The final interference takes place inside the Stokes detector and the tilt prevents the interferometer from operating below the SNL. The details of the required Stokes measurement are not shown because they depend on where the state is located on the geodesic, if one wants to have the best possible sensitivity. For an arbitrary position on the geodesic, one could split the output beams and perform simultaneous measurements of conjugate Stokes parameters, so that the sensitivity is state-independent. However, that approach is associated with a noise penalty [41, 42].

The new twist is based on something that was known already to Stokes: there is a whole range of different types of beam splitters, the coefficients of which differ in the phase shifts they introduce [43, 44]. In addition, by allowing for asymmetric splitting ratios, the action of a beam splitter can lead to any basis change. All this gives one much freedom in how one can choose the beam splitter to act, provided one tailors the phase shift appropriately. The important lesson is that it is now favorable to insert the central beam splitter in Figure 1 and arrange the rotation axis of the beam splitter such that ‘downstream’, the state before detection moves along the red geodesic which is perpendicular to the ellipsoid, as we can see in Figure 2. This improves the sensitivity beyond the SNL for any phase change *δψ* arising after the beam splitter.

The axis of the required rotation is the *S*_3_ axis, and the rotation is shown in the inset in Figure 2 as the angle 4*α*. Consider an $S_{2}^{\prime }$ parameter measurement. (The prime relates to the rotated Stokes parameter set $\left\{S_{1}^{\prime },S_{2}^{\prime },S_{3}\right\}$ , as indicated in Figure 2). The uncertainty ellipse is projected onto the $S_{2}^{\prime }$ axis, resulting in a distribution around the mean value of width $\Delta {\hat{S}}_{2}^{\prime }=\sqrt{\left\langle {\hat{S}}_{2}^{\prime 2}\right\rangle -{\left\langle {\hat{S}}_{2}^{\prime }\right\rangle }^{2}}$ . A small phase shift of *δφ* before the beam splitter moves the ellipse along the geodesic *C*_*φ*_, changing the average value of ${\hat{S}}_{2}^{\prime }$ by $\delta_{\varphi }\left\langle {\hat{S}}_{2}^{\prime }\right\rangle$ , and after the beam splitter—along the geodesic *C*_*ψ*_. The phase shift *δφ* can be detected only if the projections of the two ellipses on the $S_{2}^{\prime }$ axis do not significantly overlap; i.e., $\delta_{\varphi }\left\langle {\hat{S}}_{2}^{\prime }\right\rangle >\Delta {\hat{S}}_{2}^{\prime }$ , similarly for *δψ*. It is important here that the ellipse is rotated with respect to the measurement axis together with the geodesic *C*_*φ*_, and the squeezing angle *θ*_sq_ is fundamentally nonzero. Because of that, we cannot have the minimum $\Delta S_{2}^{\prime }$ and the maximum $\delta_{\varphi }\left\langle {\hat{S}}_{2}^{\prime }\right\rangle$ simultaneously, regardless of the angle 4*α*. There is thus no sensitivity improvement for *δφ*. On the contrary, *C*_*ψ*_ is always along $S_{2}^{\prime }$ . So, if 4*α* + *θ*_sq_ = *π*/2, the sensitivity for *δψ* is improved.

Note that interfering two equally intense squeezed light beams such that the output beams also carry equal intensity leads to entangled Gaussian beams [45], [46], [47]. The state of two light beams, separately squeezed and equally intense in the {*a*, *b*} basis, and thus separable, will lie in the *S*_3_–*S*_2_ plane [48]. However, when writing the same state in an orthogonal basis, such as $\left\{\left(a+b\right)/\sqrt{2},\left(a-b\right)/\sqrt{2}\right\}$ , it will be Einstein–Podolsky–Rosen entangled [49, 50]. This means that, if the light state lies on the *S*_3_ axis and aligned with $S_{1}^{\prime }$ , the detector has to measure the ${\hat{S}}_{2}^{\prime }$ parameter; i.e., one has to measure the photon-number difference in the $\left\{\left(a+b\right)/\sqrt{2},\left(a-b\right)/\sqrt{2}\right\}$ basis of the primed coordinates, in which the state is entangled. Thus, entanglement plays a crucial role in sensitivity enhancement, although not obvious in the Poincaré representation. While the interferometer offers sub-SNL sensitivity for any arm length difference *δψ* introduced to the right of the beam splitter (i.e., independent of where the final state is located on the red geodesic), the Stokes parameter which has to be measured to reach this maximum sensitivity does depend on where the state resides on the red geodesic.

## 3 Experimental setup

**Figure 3:**
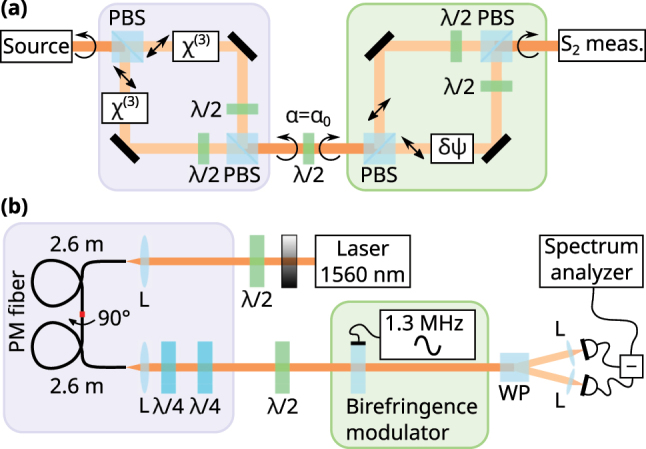
Scheme of a Kerr-squeezed interferometer. (a) General free-space scheme of a Kerr-squeezed interferometer. The polarization-squeezed state is prepared in the blue box, then the basis is rotated with a half-wave plate, and in the green box a phase change is introduced that can be measured with sensitivity below the SNL. The polarization modes in the boxes may share the same spatial mode (in this case no PBSs are used) and are drawn separately for clarity. (b) The scheme of our experimental setup. The boxes correspond to those in (a). PBS — polarizing beam splitters, L — lenses, *λ*/2 — half-wave plates, *λ*/4 — quarter-wave plates, WP — Wollaston prism.

For the experimental demonstration of this sub-shot-noise operation, we encode the two rails in Figure 1 as the diagonal and anti-diagonal linear polarizations in the same spatio-temporal mode. A general scheme to generate two equally bright Kerr squeezed states is shown in the blue boxed part of Figure 3a. A circular polarization state is created after the second polarizing beam splitter. The two polarization modes, which are drawn separately for clarity, can occupy in practice the same spatial mode. It is convenient to use a polarization-maintaining fiber [48] for that, using both its polarization modes simultaneously. The following half-wave plate at angle *α* performs a rotation in the Poincaré space around the *S*_3_ axis by the angle 4*α* (see the inset of Figure 2). Note that the action of the wave plate can be equivalently described as a rotation of the state while keeping the basis fixed. In this way, in absence of the green-boxed part, the fluctuations of an arbitrary linear combination of ${\hat{S}}_{1}$ and ${\hat{S}}_{2}$ Stokes parameters can be directly measured using a fixed ${\hat{S}}_{2}$ balanced detector. With a proper choice of the wave plate angle *α* = *α*_0_, such that 4*α*_0_ + *θ*_sq_ = *π*/2, one can observe a reduction in fluctuation amplitude; i.e., squeezing. Usually, this half-wave plate is considered part of the Stokes parameter detector, however, in our scheme, it has a more important function.

It turns out that when *α* = *α*_0_, the wave plate acts exactly as the required beam splitter in Figure 1, and any phase change between rails *a* and *b* ‘downstream’ from the half-wave plate can be measured with quantum enhanced sensitivity. Thus, in the green boxed area of Figure 3a, the phase difference *δψ* is introduced, moving the ellipse in the direction perpendicular to its major axis and along the great circle in the $S_{2}^{\prime }-S_{3}$ plane simultaneously (see Figure 2). The sensitivity of *δψ* measurement is better than what we can get with a coherent state. Again, the two polarization modes do not have to be spatially separated; however, it might be favorable to do so in a full-scale free-space interferometer. It is important that in the case the polarizing beam splitters are used to separate the modes spatially, additional vacuum polarization modes entering the setup through dark ports of PBS do not interfere with bright modes and do not destroy squeezing. Additional losses are introduced only by the optical elements and by choosing them properly, hardly any squeezing should be lost. Note that the choice of the measurement and the rotation axes are not fixed, the only requirement is that the minor axis of the uncertainty ellipse should be aligned with the measurement axis, while the major axis should be aligned with the rotation axis.

The specific scheme of our proof-of-concept setup is shown in Figure 3b. We used our new robust setup [34] to generate polarization squeezed states using the Kerr effect in a polarization-maintaining (PM) fiber. The source emitted 200 fs (full-width half-maximum) shot-noise-limited pulses at a central wavelength of 1560 nm with an 80 MHz repetition rate. The pulse energy was attenuated to 160 pJ.

These pulses propagate in the fiber as solitons, which to a good approximation provide a well-defined single spatio-temporal mode [55]. Nevertheless, the Stokes operators defined above can be generalised to a multimode description [48] and the discussion in Section 2 applies despite the pulsed nature of the light used. Frequency-dependent effects may lead to a broadening of the effective squeezing ellipse. As a result of the soliton dynamics, the nonlinear processes persist throughout the fiber.

A diagonal polarization with respect to the fiber birefringence axes was set to create two equal pulses in the two polarization modes of the PM fiber. The fiber (3M FS-PM-7811, 5.2 m) was split into two halves, which were spliced back with a 90° turn around the fiber axis to match the group delays of the two pulses. Two quarter-wave plates were used to adjust the polarization to be circular, and then a half-wave plate was installed to align the minor axis of the ellipse to the $S_{2}^{\prime }$ axis (in the inset of Figure 2, *α* = *α*_0_). The squeezing was more than 5.0 dB.

After that, a glass plate with a mounted piezoelectric transducer was used to introduce a phase difference between the two diagonal polarization modes. For various applications, this plate can be replaced with a setup shown in the green box in Figure 3a that spatially separates the modes. The transducer exerts stress on the plate to introduce a small birefringence with the axes at 45° to the horizontal. Thus, the plate serves as a variable wave plate. It is modulated at 1.3 MHz, and in turn modulates the ${\hat{S}}_{2}^{\prime }$ Stokes parameter. Next, the ${\hat{S}}_{2}^{\prime }$ parameter is measured using a balanced detection scheme. The ${\hat{S}}_{2}^{\prime }$ parameter in the diagonal basis of the birefringence modulator is the difference in photon number in vertical and horizontal polarization modes. A Wollaston prism is used to separate these polarizations, and the respective optical power is individually detected with two high-quantum-efficiency photodiodes. The photocurrents are then amplified, subtracted, and fed to an electronic spectrum analyzer (ESA, Agilent E4411B). There, the signal spectrum is measured between 1.2 and 1.4 MHz with a resolution bandwidth of 10 kHz and a video bandwidth of 30 Hz. Without modulation of the ${\hat{S}}_{2}^{\prime }$ parameter, this spectrum represents its fluctuations with intensities of spectral components proportional to $\Delta S_{2}^{\prime }$ . When ${\hat{S}}_{2}^{\prime }$ is modulated, an additional signal at the modulation frequency appears, proportional to $\delta_{\psi }\left\langle {\hat{S}}_{2}^{\prime }\right\rangle$ .

**Figure 4:**
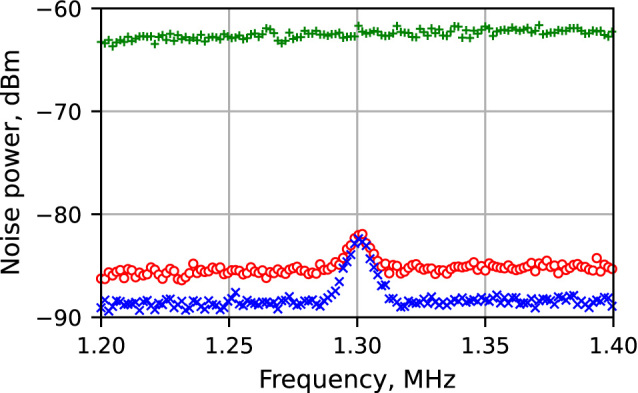
Spectra of the Stokes parameter measured with the birefringence modulator in action: bottom (blue crosses) – modulating the squeezed Stokes parameter, middle (red circles) – modulating a coherent state of the same power (background equivalent to shot-noise level), top (green pluses) – modulating the anti-squeezed Stokes parameter for comparison. The uneven background level is due to the amplifier response.

A typical spectrum with enabled transducer is shown in Figure 4 in blue crosses, in comparison with similar setups: (1) when using and modulating a coherent state and (2) when modulating and measuring the anti-squeezed component (4*α* = −*θ*_sq_). The peak in the center is the signal, while the background is the quantum noise. The signal power is essentially the same using squeezed and coherent states, while the noise level is much lower when squeezed light is used. The signal-to-noise (SNR) ratio is enhanced by 4.0 ± 0.5 dB with the use of the squeezed state, proving the efficiency of the proposed scheme. When the anti-squeezed Stokes parameter is modulated and measured, the signal is lost in the noise. We note that in the absence of optical losses the SNR enhancement in the proposed scheme should be equal to the squeezing amount.

Table 1 summarizes experimental demonstrations of optical interferometers with quantum-enhanced sensitivity in comparison with the result reported here. While all other demonstrations used a *χ* ^(2)^-nonlinearity, our work is the first involving a *χ* ^(3)^-nonlinearity. We expect that this novel scheme will go through a similar development with remarkable improvements as seen for the *χ* ^(2)^ schemes. The inherent simplicity of the *χ* ^(3)^ or Kerr squeezing could well be an advantage for applications. We believe that the proposed interferometer concept is well compatible with nanophotonic waveguide integrated platforms based on materials with high Kerr nonlinearity and low losses, such as silicon nitride, in which the key building blocks of interferometers are available (waveguide splitters and combiners, etc.), and the possibility of quadrature squeezed light generation was demonstrated [56, 57].

**Table 1:** Squeezed-light-enhanced interferometers beyond the shot-noise limit.

Reference	Parametricdown-conversion	Optical Kerr effect	Setup	Detection frequency	Observed sensitivityenhancement beyond SNL
Xiao 1987 [17]	X		table-top	1.6 MHz	3 dB
Grangier 1987 [18]	X		table-top	400 kHz	2 dB
Goda 2008 [51]	X		laboratory	50 kHz	3.2 dB
Acernese 2019 [22]	X		Advanced Virgo	3 kHz	3.2 dB
Lough 2021 [52]	X		GEO 600	6.4 kHz	6.0 dB
Heinze 2022 [53]	X		table-top	4 MHz	10 dB
Zander 2022 [54]	X		table-top	4.9 MHz	10.5 dB
This work		X	table-top	1.3 MHz	4 dB

In our experiment, the SNR enhancement is a bit less than the directly measured squeezing, due to Fresnel reflections from the faces of the birefringence modulator. It is possible to reduce these losses to a negligible value in our experiment using anti-reflection coating, as well as in any full-scale interferometer that follows the general scheme in Figure 3a by the use of properly selected optical elements.

The proposed way to enhance the interferometer sensitivity can, in principle, be implemented in the low-power regime in an all-fiber format and operate directly at an eye-safe wavelength. This does not require any intermediate second harmonic generation stages unlike the approach recently demonstrated for a quantum-enhanced low-power interferometer [54].

A final remark concerns the possibility of going beyond the SQL in the new scheme. For the traditional interferometer, the two ports are fed by one intense coherent beam and one squeezed vacuum beam. When increasing the light power in the coherent beam enough and using reflecting mirrors in the two arms, one ultimately reaches the SQL beyond which light power fluctuations dominate [2]. As discussed in the Introduction, by appropriately correlating the amplitude and phase noises at the vacuum input port one can go beyond the SQL [6], [7], [8], [9]. A related improvement by correlating different noise contributions is also expected here for the Kerr squeezing enhanced interferometer fed with two intense squeezed beams at the two input ports, as in the scenario discussed above (see page 218 in Refs. [44]).

## 4 Conclusions

In conclusion, we report the first demonstration of measuring optical path length differences interferometrically with sensitivity well below the SNL using Kerr-squeezed light. The new type of enhanced-sensitivity interferometer with a robust and reliable source of pulsed Kerr-squeezed light, eye-safe wavelength near 1500 nm, and low average power operation might be attractive for applications. The key feature of the setup presented here allows one to reach sub-SNL sensitivity also with Kerr squeezing by introducing a basis change inside the interferometer between the sources of nonlinearity and the introduction of a phase difference.
